# Orderliness of Visual Stimulus Motion Mediates Sensorimotor Coordination

**DOI:** 10.3389/fphys.2018.01441

**Published:** 2018-10-11

**Authors:** Joshua Haworth, Nicholas Stergiou

**Affiliations:** ^1^Department of Kinesiology, Science and Learning Center, Whittier College, Whittier, CA, United States; ^2^Department of Biomechanics, University of Nebraska Omaha, Omaha, NE, United States; ^3^Department of Environmental, Agricultural & Occupational Health, College of Public Health, University of Nebraska Medical Center, Omaha, NE, United States; ^4^Division of Biomechanics and Research Development, College of Education, University of Nebraska Omaha, Omaha, NE, United States; ^5^Department of Biomechanics, University of Nebraska Omaha, Omaha, NE, United States

**Keywords:** biological motion, eye tracking, smooth pursuit, coupled systems, determinism, gaze, posture

## Abstract

We explored the coupling of gaze and postural sway to the motion of a visual stimulus, to further understand sensorimotor coordination. Visual stimuli consisted of a horizontally oscillating red dot, moving with periodic (sine), chaotic, or aperiodic (brown noise) temporal structure. Cross Recurrence Quantification Analysis (cRQA) was used to investigate the coupling between each measured signal with the time series of the visual stimulus position. The cRQA parameter of percent determinism indicated similar strength of coupling of gaze with either periodic or chaotic motion structures, yet weaker coupling to aperiodic stimulus motion. The cRQA parameter of Maxline indicated a particular affinity toward chaotic motion. Analysis of postural coupling supports the idea that the complex periodicity of body sway affords interactivity with non-simple environmental dynamics. These results collectively strengthen the argument that chaos is an invariant and beneficial feature of biological motion, a feature which may be critical for immediate and robust coordination of the self with the environment and other environmental agents.

## Introduction

Humans exhibit oscillatory dynamics on many time scales, from sleep/wake cycles to breathing to regulation of posture. Even the routine of trips to the grocery must be repeated after some time has passed since the last visit. These are all processes which can be discretized, allowing their iterations to be viewed as single events. In actuality, however, each individual event truly occurs within series, with potentially critical interdependencies between iterations, making each event part of a more general continuity. To ensure success in this complex world, individuals must possess some means by which to coordinate the memories they have about previous events along with predictions about future events, all in line with the real-time ‘now’ which they are experiencing (Spivey, 2007). Our goal in this paper is to provide in the introduction a contextual motivation for an experiment that explores the role of the complexity of stimulus orderliness as a mediator of sensorimotor coordination, and then through empirical analysis to provide further discussion.

Sejnowski (2010) discussed the propensity of both monkeys and humans to learn optimal strategies in the face of complex problems. Moreover, these behaviors have been replicated in reinforcement based simulations which demonstrate the emergence of solutions given sufficient time to generate experience. This concept is further extended to observations of how children seem to learn so naturally, through simple attempt repetitions and imitation play (Meltzoff et al., 2009). This discovery learning approach has also been discussed by Berthier et al. (2005) where they have demonstrated the efficacy of a fully unsupervised model to generate reaching behavior similar to that which we would expect from a typical human child. In contrast to a supervised model, where ‘correct’ strategies are instructed, unsupervised models foster the self-discovery of the dynamics of the internal and external environments.

However, it is not just the status of the agent and environment that serve to inform motor strategy development. Thelen and Smith (1994) showed extensively that interaction dynamics serve a large role in the development of motor strategies. Both internal and external constraints can serve to limit our coordination space, reducing the number of potential movement strategies that are available to choose from Todorov and Jordan (2002) extended that variability in movement behavior is ubiquitous, leading further to the conclusion that pre-planned regulations of movement strategies could not possibly be effective. Instead, they demonstrate that it is through real-time feedback (and its optimal control) that purposeful actions are realized. Such a control process should afford easy consideration to even the most complex of experienced dynamics. Albeit, extensive practice might yet be necessary in order to ‘learn’ the most effective uses of such feedback. We see here a fundamental shift in what it takes to excel in the world, from a strategy of *learn the right way* to *learn the right coordination*. This paradigm shift could have massive impact on how we continue to organize learning environments.

In light of this new perspective, it becomes clear that the quality of experience is one of utmost importance. Here, we do not mean the ‘goodliness’ type of quality, but instead refer to factors of organization of the experience within space and time; essentially, the ‘orderliness’ of the learning experience. Sprott (2013) has shown that the inclusion of chaotic dynamics is specifically beneficial to learning in artificial neural networks, suggesting that the benefit of chaos is its core nature of deterministic variability. This flexibility affords the exploration of many combinations of degrees of freedom (potential strategies), while maintaining structural similarity that can be revisited in future iterations of practice. This point echoes the arguments of many others in describing important aspects of play en route to a successful motor repertoire (Siegler, 1996; Adolph and Joh, 2009; Siegler et al., 2010). The suggestion that we may utilize chaos as a means to optimize our learning strategies is quite an interesting notion. This is especially true in light of other recent work describing the general inherence of chaos (and complexity) in the optimization of human behaviors, including movements (Haworth et al., 2013). Further work by Ali et al. (2007) affirmed this idea of inherent chaos in human movement by showing that algorithms for automated motion tracking are more proficient at capturing biological motion when they are set to attend to chaotic motion structures.

The notion of chaos is extendible even to the domain of humanoid movement behavior. Schaal (2007), discussed the role of behavioral variance in helping to make the actions of robots more human-like in order to lead to greater social tolerance. Interestingly, people seem to be quite sensitive to the apparent rigidity of robots. Something about the disproportionate predictability of a robot’s motions, relative to a typical person’s, makes it a generally unnatural and uneasy visual experience. The assertion that optimum dynamics should include some factor of variance (i.e., from chaos) is certainly interesting, and apparently also quite practical. Duran et al. (2007a,b, 2008) showed that it is possible to develop real-time, dynamic smooth pursuit behavior in robotic systems, using a coupled chaotic systems approach. This tracking behavior is readily responsive to known and novel objects, and their motion trajectories. Moreover in this approach, vision is shown to be sufficient to inform the self-organization of the motion of the postural coordination necessary for object tracking; i.e., neck muscle activations. However, much is yet to be discovered in this domain of sensorimotor connectivity, particularly with regard to the vast expanse of interaction dynamics that are necessary for success in the world.

It should be noted here, that not all environments are actually ultimately complex. Often, either by natural order or imposed organization, we find ourselves operating in a circumstance which is out and out routine. Other times, we may find our operations to be within an environment which truly has no organizational process to it, whatsoever. That which is truly remarkable is that our ability to organize our own behavior is almost ubiquitously robust against these environmental variances. Continued cooperation between human movement scientists and roboticists would surely be mutually beneficial, heading toward elegant descriptions of the way by which persons and machines can interact with environments that range across periodic, through chaotic, even toward completely random orders. As human movement scientists, we make the assertion that an individual’s primary means toward ‘success’ is through effective coordination of their actions within the organization of the world. This requires competency in the production of purposeful movements, but even more so a competency in determining the dynamics of the world within which those actions are to take place.

Several modern techniques have emerged that offer quantitative strategies for assessing qualities of synchronicity between connected systems. Connectivity analysis is often used to study rhythmic neuronal interactions to identify inter-areal synchronization within the brain using techniques such as conditional Granger causality index and partial transfer entropy (Bastos and Schoffelen, 2016). In the fields of action perception and ecological psychology, cross recurrence quantification (cRQA) has been used extensively to elucidate human behavior coupling between two persons or between a person and an environmental stimulus. Common applications include evaluation of social interactions (Richardson et al., 2008; Fusaroli et al., 2014; Davis et al., 2017), conversational dynamics (Dale and Spivey, 2005, 2006), head motions during conversation (Paxton and Dale, 2017), postural coordination (Shockley et al., 2003; Shockley, 2005; King et al., 2012), and eye movements (Richardson and Dale, 2005; Richardson et al., 2007), as well as posture and gaze response to visual stimulus motion complexity (Haworth et al., 2015, 2016).

Thus, we seek in the current work to better understand human-environment interaction by testing the role of the complexity of stimulus orderliness as a mediator of sensorimotor coordination. We sought to find a better understanding through this experiment of the sensitivity and responsiveness of human vision and posture, in response to rhythmic, chaotic, and random motion. Our analysis utilizes percent determinism and maxline outcomes from cross recurrence quantification to evaluate qualities of coupling between each of these systems, as these have been shown previously to uncover interesting aspects of engagement dynamics (Haworth et al., 2015, 2016). We intend to conclude with the assertion that persons are able to observe and respond to a full spectrum of motion dynamics, maintaining particular affinity for chaos.

## Materials and Methods

### Participants and Procedures

Fourteen healthy young adults (4 male and 10 female, age 29.8 ± 10.5 y, height 1.638 ± 0.1 m, and weight 67 ± 14.2 kg) each participated in a single individual session for data collection. Synchronous eye movement and standing posture recordings were taken while a moving point-light stimulus was displayed on a large monitor in front of the participant. FaceLab 4.5 (Seeing Machines, Acton, MA, United States) eye-tracking equipment was used to track eye movements. An AMTI force platform (Advanced Mechanical Technology Inc., OR6-7, with MSA-6 amplifier) was used to record center of pressure (the projection of the body’s center of mass onto the surface) throughout each trial. Trials were managed through custom software designed in LabView (National Instruments, Austin, TX, United States), including software synchronization of the data from the eye-tracker and the force platform, as well as the display of the visual stimulus. All data was collected at 50 Hz, as this was the highest common frequency available amongst the set of equipment. Additionally, 50 Hz sampling provides 20 ms resolution of each measured behavior, which is sufficient to observe the dynamics of both postural sway and smooth pursuit eye movements. We purposefully steered away from stimulus oscillation velocity/frequency that could provoke saccade or rapid postural perturbation.

**FIGURE 1:**
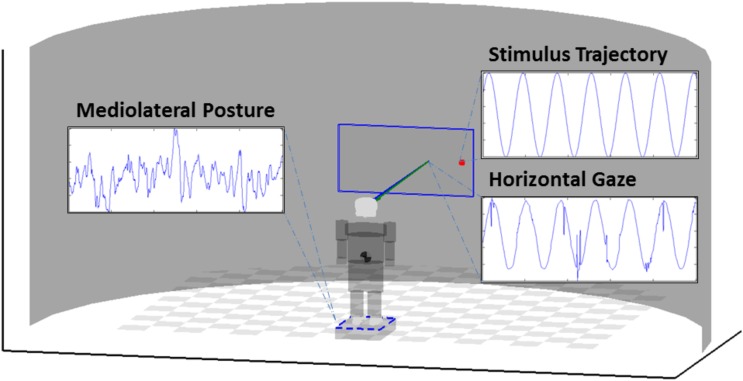
Diagram of the experimental setup. Participants stood atop a force platform in comfortable, self-selected stance. Eye-tracking equipment is affixed to the monitor stand, and positioned to capture gaze response during stimulus presentation. Example time series shown are from the Sine condition.

The displayed stimulus (a red dot, 25 pixel radius) was presented on a 55″ 1920 × 1200 pixel LCD monitor, moving according to a predefined motion trajectory (sine, chaos, and brown noise) with an update in position occurring at a rate of 50 Hz. Trials lasted for 5 min each to ensure the capture of adequate lengths of data, with condition order randomized for each participant. Participants were given the instruction to stand quietly and attend to the motion of the stimulus until the end of the trial, as indicated by the investigator. Room lights were dimmed, and conversation was held to a minimum throughout each trial. However, participants were allowed to speak and move about freely in the time between conditions. Grid markings on the surface of the force platform were used to realign the feet to ensure a similar stance between each condition. **Figure 1** provides a diagram of the setup. Informed consent was obtained prior to all experimental procedures, as approved by the University of Nebraska Medical Center Institutional Review Board.

### Stimulus Presentation

Stimulus motion animations were constructed such that the position of the stimulus was updated at 50 Hz (above perceptive threshold of object motion), with each new point defined to follow one of three main signal structures; sine, chaos, and brown noise. These particular signals were selected, as they span the domain of ‘orderliness.’ Sine exhibits perfectly redundant order, brown noise exhibits stochastic or non-redundant order, with chaos exhibiting a dynamic and deterministic mixture of redundancy and non-redundancy.

Each signal is comprised of 15,000 data points updating at 50 Hz, to provide 5 min of continuous stimulus motion. Stimulus time series were constructed using embedded and custom algorithms in Matlab (MathWorks, Natick, MA, United States) and saved in data files on the computer. These data series were accessed and displayed through the main Labview application during each trial. The sine signal was generated using the sin() function in Matlab. Single sinusoidal motion represents the simplest oscillation, such as a frictionless clock pendulum, expressing perfect redundancy. The chaos signal was produced from a model of the motion of a double pendulum, which has recently been shown to successfully model the dynamics of human posture (Suzuki et al., 2012). The free rotations of a two segment linkage are sufficient to afford chaotic dynamics (Shinbrot et al., 1992), which appear in the hip strategies expressed by their model. The *x*-axis position of the distal segment of our custom model was extracted and used to produce the chaos signal. Surrogate testing via phase randomization (Theiler et al., 1992) was used to further the confidence that the generated signal exhibited chaotic dynamics. The brown noise signal was generated by the iterative addition of a random perturbation to the original point position. White noise truly represents purely stochastic nature; however, it would be impossible to follow such a structure with smooth pursuit eye movements. Smooth pursuit requires continuity in the motion of the tracked object. Brown noise in essence is the integral of white noise; maintaining the stochastic nature, while also presenting sufficient continuity to be tracked by smooth pursuit eye movements. Although contentious, human posture has previously been touted to express Brownian motion (Collins and Deluca, 1994); lending confidence to our approach that the Brown Noise stimulus is available for integration in sensorimotor coordination. This spectrum of particular signals provides us with an access to investigate how individuals might manage their gaze and posture in coordination to various motions; particularly on the aspect of orderliness.

### Data Processing

Gaze and postural data were recorded at 50 Hz, throughout the entire 5 min duration of stimulus condition presentation. Gaze data was recorded as the on-screen pixel coordinate at which the participant was looking at each time point throughout the trial. Center of pressure was recorded as the measure of posture. For both signals, only the horizontal component of motion was further considered, as the stimulus signal was set to move only in the horizontal direction. To avoid the influence of novelty, the first ten seconds of data was eliminated. Only the subsequent 2 min of data were further processed, to reduce the possibility that fatigue might interfere with the quality of the analyses. Cross recurrence quantification analysis (cRQA) was used to assess coupling of gaze (Gaze) and posture (COP) to the stimulus, separately, as well as between gaze and posture to gauge sensorimotor coupling (SensMot). Metrics of SensMot provide additional novelty to this work, as it provides a first effort toward a ‘direct’ assessment of the continuous relationship between gaze and posture during an attention driven smooth pursuit task.

Outcome metrics from cRQA were calculated using custom Matlab software (MathWorks, Natick, MA, United States) adapted from those provided by the Perceptual-Motor Dynamics Laboratory at the University of Cincinnati, are described in further detail below (Shockley et al., 2002; Shockley, 2005). Prior to conducting cRQA, gaze data was pre-processed to remove zero (0) values that were recorded during collection from a small number of files; much smaller than 0.01% of data in each file. These data were registered by the eye-tracking software during samplings when the eyes were unable to be imaged for position analysis. This occurred in our case, when persons had an exceptionally long blink. These values were removed from the time series, and replaced using a 5th order cubic spline (Matlab, *interp1* function).

#### Cross Recurrence Quantification Analysis

Webber and Zbilut (1994) developed recurrence quantification analysis to assess the structure of the temporal evolution of a behavior through the associated measured time series. This process includes embedding a time series into its respective multi-dimensional phase space (Takens, 1981), creating a recurrence matrix (recurrence plot), and then applying various pattern matching algorithms to uncover the underlying dynamics. Later, Zbilut et al. (1998) expanded this technique to include applying these pattern matching approaches to recurrence matrices generated from two separate time series embedded in similarly dimensional phase space. This approach proved useful for uncovering mutual dynamics between the two time series, and has since been used to describe coupled oscillators in many various disciplines, including the coordination of chaotic oscillators (Shockley et al., 2002). Particularly of interest to the current work are the applications of cRQA to discover metrics of coordination of coupled biological rhythms (Richardson and Dale, 2005; Richardson et al., 2008).

In order to conduct cRQA, each time series must be unfolded into a similar multi-dimensional phase space. This is accomplished using parameters of delay and embedding dimension, which are calculated from average mutual information (AMI; Fraser and Swinney, 1986) and False Nearest Neighbors (FNN; Abarbanel, 1996) algorithms. We used values of 22 and 10, respectively, as these were the group averages after passing each dataset through the above algorithms.

**FIGURE 2:**
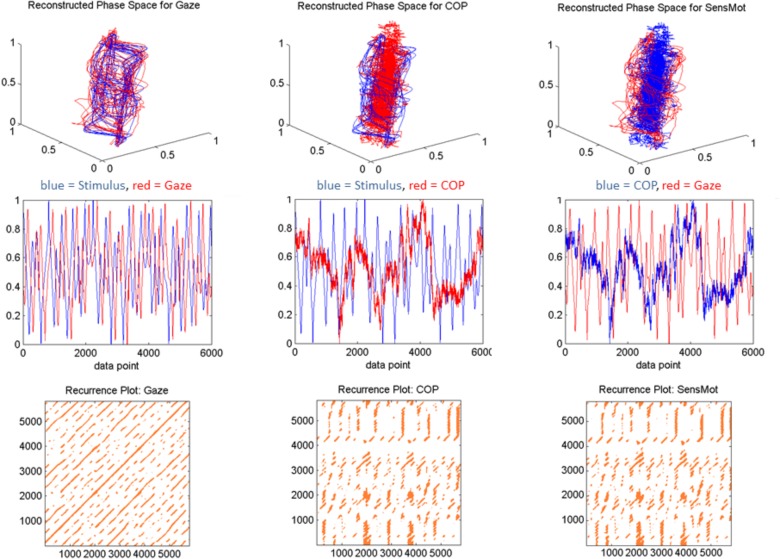
Sample data from a single, representative participant in response to the chaotic stimulus motion. The first three viewable dimensions of the two signals are shown in the first row, with the single-dimensional measured signal in the middle row. The bottom row shows the recurrence plot which indicates the points of coordination, across trial duration, in phase space. This order is repeated for each of Gaze, COP, and SensMot in separate columns as labeled. All data are presented unit normalized spatially and from index value 0 through 6000, corresponding to 2 min of trial duration at 50 Hz.

In order to achieve outcome data that was both representative of the time series’ dynamics, and yet also reasonably comparable, we chose to set a fixed recurrence value instead of radius value; as has been previously suggested (Shockley, 2005). Otherwise, we found that the determinism saturated, and no effective interpretations could be made of the recurrence plots (which by the eye, clearly showed differences; see **Figure 2**, bottom row). We set percent recurrence at 5%, such that we would subsequently evaluate recurrent lines under parameters that are more similar across the three stimulus conditions. Minline represents the shortest duration (in data points) within which the two signals are sequentially recurrent that will be considered in subsequent computations. Minline was set in our experiment to 25, representing duration of 0.5 s as a minimum threshold to be considered as a recurrent line. This value was chosen based on the logic that smooth pursuit and saccadic eye movements can both occur in shorter time spans, but the saccades would not last longer. Additionally, early runs of sampled data suggested that this value would provide more stable and comparable outcome measures across the three conditions.

Outcome measures from cRQA to be considered here include percent determinism and maxline. These outcomes each provide a unique description of the dynamics available from the cross recurrence plot as they have previously been shown to elucidate dynamical coordination in tasks similar to those used in the current experiment (Shockley et al., 2002, 2003; Richardson et al., 2008). Percent determinism is the ratio of recurrent points that form lines, divided by the total number of recurrent points; reported from 0 to 100%. If every point of recurrence between the two signals is part of a bout of continuous coordination (minimum of 25 points to form a line), percent determinism would report as 100%. It is possible that none of the recurrent points are part of a continuous coordination (line), in which case percent determinism would report as 0%. Maxline is the length of the longest line formed by recurrent points, expressing the extent of coupling between the two signals; reported in number of data points. Larger values of maxline indicate longer bouts of continuous coordination between the compared behaviors. Data is collected at 50 Hz, so each increment of 50 data points for maxline represents 1 s of signal coordination.

#### Statistical Analysis

Separate one-way, repeated measures ANOVAs (within subject; comparing periodic, chaotic, random conditions) were conducted to test percent determinism and maxline across these three stimulus conditions; for each of Gaze, COP, and SensMot. *Post hoc*, dependent *t*-tests were used to identify where differences occurred. Statistical tests were conducted using IBM SPSS Statistics software (IBM Corporation, Armonk, NY, United States, Version 18), with an alpha set at 0.05.

## Results

**FIGURE 3:**
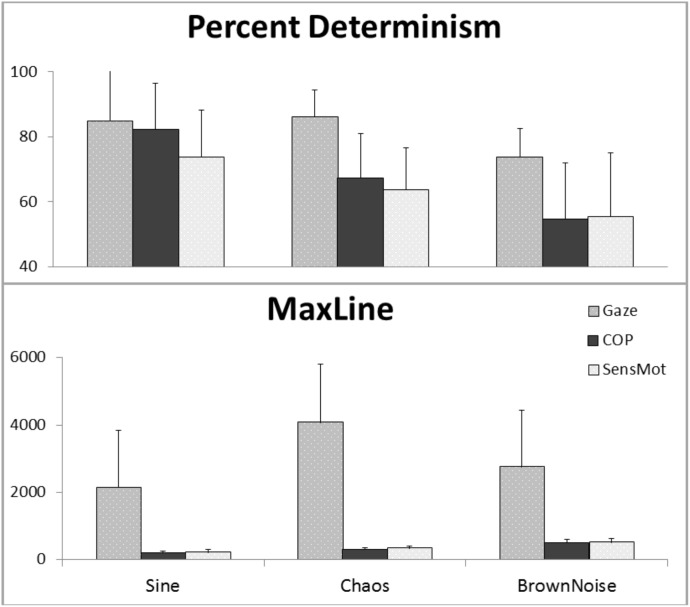
Results of cRQA, showing coupling for Gaze and COP to stimulus motion (Gaze and COP, separately) and in relation to one another (SensMot), across three types of stimulus motion.

Results are reported below, separately for each of Gaze, COP, and SensMot. A graphical view of the results can be found in **Figure 3**, with a listing of the pairwise *t*-values in **Table 1**. Additional analyses were conducted, with proximal parameterizations to those reported, in order to verify the robustness of the findings. In each case, similar results and trends were found as those reported here.

**Table 1:** Mean values of the group (*n* = 14) for each outcome measure, under each stimulus condition.

Stimulus signal	Gaze	*T*-tests		COP	*T*-tests		SensMot	*T*-tests	
Percent Recurrence
**Sine**	**85**	Sine ⋅ Chaos	0.3371	**82.3**	Sine ⋅ Chaos	0.0049^∗^	**73.8**	Sine ⋅ Chaos	0.0315^∗^
**Chaos**	**86.2**	Chaos ⋅ Brown	0.0000^∗^	**67.4**	Chaos ⋅ Brown	0.0000^∗^	**63.7**	Chaos ⋅ Brown	0.0119^∗^
**Brown Noise**	**73.8**	Brown ⋅ Sine	0.0026^∗^	**54.7**	Brown ⋅ Sine	0.0003^∗^	**55.4**	Brown ⋅ Sine	0.0116^∗^
MaxLine
**Sine**	**2137**	Sine ⋅ Chaos	0.0005^∗^	**208**	Sine ⋅ Chaos	0.0002^∗^	**217**	Sine ⋅ Chaos	0.0004^∗^	
**Chaos**	**4071**	Chaos ⋅ Brown	0.0305^∗^	**308**	Chaos ⋅ Brown	0.0000^∗^	**333**	Chaos ⋅ Brown	0.0001^∗^	
**Brown Noise**	**2741**	Brown ⋅ Sine	0.2134	**499**	Brown ⋅ Sine	0.0000^∗^	**511**	Brown ⋅ Sine	0.0000^∗^	


*Values in bold indicate the mean of the group for each measure (percent recurrence or maxline) of each behavior (Gaze, COP, or SensMot) in response to each stimulus type (Sine, Chaos, or Brown Noise). *T*-tests were conducted pairwise, as indicated, with a threshold at ^∗^*p* < 0.05 for significance.*

### Gaze

The one way ANOVA for percent determinism resulted in significant differences (*p* < 0.001). The *post hoc* analysis (**Table 1**) showed that percent determinism was similar for Gaze in response to the Sine and Chaos signals, but was lesser in response to Brown Noise in comparison to the Sine or Chaos signal. For maxline, ANOVA again found significant differences (*p* = 0.04). *Post hoc* analysis showed maxline of Gaze was largest during the Chaos condition, indicating significantly longer bouts of coordination with Chaos than with either Sine or Brown Noise signals.

### COP and SensMot

The one way ANOVA for percent determinism resulted in significant differences for both COP (*p* < 0.001) and SensMot (*p* = 0.004). *Post hoc* analysis (**Table 1**) showed similar patterns of response to the three conditions for COP and SensMot. Rates of coordination were highest in response to the Sine signal, and lowest in response to the Brown Noise signal. For maxline, significant differences were found for both COP (*p* < 0.001) and SensMot (*p* < 0.001). Again, *post hoc* analysis (**Table 1**) showed similar patterns for COP and SensMot. Shorter duration coordination was found in response to the Sine signal, while longer duration coordination was found in response to the Brown Noise signal.

## Discussion

We found that our results support the assertion that persons are able to observe and respond to a full spectrum of motion dynamics. Percent determinism shows that gaze had similar propensity to track Sine and Chaos, indicating an ability to maintain coupling with these signals throughout the trial. Gaze in response to Brown Noise had a significantly lower percent determinism, suggesting a weaker coupling with this motion structure. It is worth noting, though, that above 70% determinism does indicate an ability to coordinate with the random signal, yet in contrast with the other motion structures tends to not couple as strongly. Our question here is whether this reduced coupling to randomness is representative of a system limitation, or the demonstration of preference. Regardless, these results suggest that gaze behavior is proficient in response to a variety of motion structures, and is robust to motion variation of chaotic order. Actually, in looking at maxline data, it appears that persons expressed a particular affinity for chaotic motion.

Maxline represents the longest duration (in data points) within which the two signals are sequentially recurrent. With regard to gaze behavior, we contend that this measure stands as a proxy for the attention span, or ability combined with interest, to maintain stimulus following. Our results indicate that the tendency for coupling is highest in the chaos condition, and similar for the sine and brown noise conditions. In fact, persons coupled with chaos for nearly twice the duration of either of the alternatives. Recall also, the inherency of chaos in biological animacy (Haworth et al., 2013). In turn, these data may lend new insight to how we might understand the interpersonal coordination that has been described previously (Shockley et al., 2003, 2009).

Further, and as suggested above, we feel that the result of gaze maxline being the highest during the chaos condition is very much tied to attention. This interpretation is certainly a bit speculative, as we did not test or measure anything directly explicating attention as an outcome or as a mechanism. However, it is an interesting and reasonable interpretation. Coordinating gaze to the stimulus motion affords continued accrual of information which could be used to predict its future position. In the case of the Sine stimulus, the repetitive nature of the motion trajectory dispels the benefit of highly coupled gaze. It is just a simple periodic rhythm, identifiable in a short viewing period. Following a prediction of periodicity would require only intermittent viewing to confirm the prediction, and continue as such. This interpretation helps to clarify the higher percent determinism value that we found in response to the Sine stimulus. This may represent the viewer continuing to ‘come back’ to a coordination state in order to verify the constancy of the periodicity assumption that was drawn after a few cycles.

In the case of Gaze response to the Brown Noise stimulus, two unresolvable possibilities exist; either the gaze coupling is so difficult that it cannot be maintained for such longer durations, or the information gained from coupling is poor enough to dispel interest in the continuation of coupling. Although we cannot make a certain conclusion, we look to the gaze behavior in response to the sine motion and our previous interpretation. In that case, the percent determinism was high while the maxline was reduced, suggesting that an intermittent attention strategy had been adopted. In the Brown Noise condition, both metrics were depressed. We take this to indicate a generally reduced attention to this particular signal structure. We thus conclude that the high maxline in the chaos condition indicates a behavior of preference, and not one of limited ability under the other two conditions. In other words, there seems to be a motivation for sustained attention to the chaotic motion structure, which we speculate is based from an implicit awareness of its utility. Ward and West (1998) found that persons were able to learn the underlying dynamic of a particular chaotic process, and then proceed to generate number sequences which contain that dynamic. Considering this along with evidence of the invariance of chaos in biological motion (Ali et al., 2007; Haworth et al., 2013) highlights the value of our observed ability to coordinate with such complex motion structures.

Interestingly, with regard to COP, percent determinism decreased across each of the three conditions. More consistent coupling was found to the Sine signal, while less consistent coupling was found to the Brown Noise signal. It is possible that this is an effect of the inherent redundancy of the stimulus motion signal, itself. Postural coordination with a less redundant signal (Brown Noise) would likely result in less consistently recurrent behavioral patterns. However, the observed trend is actually opposite when we look at maxline, which indicates longest duration couplings to the Brown Noise signal and the shortest to the Sine signal. Given that the two metrics are independent of one another; this inverse relationship is not typically seen in this type of analysis. Thus, we believe it to be a behavioral and not a computational phenomenon. For a more complete understanding, we should consider the natural rhythmicity of posture. All accounts report postural sway to exhibit at least ‘noisy’ sinusoidal motion (Jeka and Kiemel, 2004). However, reports also describe posture to demonstrate Brownian motion (Collins and Deluca, 1994); and more recently, posture has been modeled to contain inherent chaotic structure (Suzuki et al., 2012). Without trying to resolve which of these accounts is more accurate, we highlight that each of them presents the case that human posture requires a more complicated model than simple sinusoidal rhythmicity. Given this, our results come into better focus in suggesting that posture coordinates more so with non-rigidly periodic motion structures.

Analysis of SensMot results in the same pattern of behaviors as was found for COP. Several interpretations appear with respect to these results. One is that the coordination of gaze to stimulus motion was sufficiently high that we would not expect dramatically different coordination of posture to stimulus and gaze. Unfortunately, this deflates somewhat the additive value of SensMot to a study which already compares gaze and posture separately. However our results do support its use as a stand-alone metric of sensorimotor coordination, which could cut data preparation and processing time in half without significant loss of information. Separately, given the similarity of results between the COP and SensMot, we speculate that the postural dynamics seem to govern the outcomes of SensMot coordination. This seems to be a bit unexpected, as the assumed information flow of the experience is from the motion of the stimulus, through the sensation/perception of its motion, to the resultant reorganization of posture. It is curious how postural dynamics could weigh more heavily in the coordination of eye and body movement if they are at the end of the information flow. Possibly, postural dynamics do have some regulative influence on the nature of sensorimotor coordination. Further research could explore the temporal resolution of the identified coordination patterns, and seek to provide additional clarity to this interpretation.

## Conclusion

Our results corroborate with previous work testing sensorimotor coupling to environmental dynamics (Stoffregen et al., 2000, 2006, 2007; Kay and Warren, 2001; Giveans et al., 2011). We have added with the current experiment, explicit evidence that these couplings are robust in the presence of chaotic motion structures of stimulus motion. This opens the way for future research to be conducted into the robustness of these findings, and the expanse of chaotic oscillators to which we are able to couple in an effective fashion. Further, we anticipate the application of this finding in the creation of therapeutic modalities that may seek to positively affect the dynamics of sensorimotor coordination in clinical populations. Lingering questions remain, however. Is attention to chaos a ubiquitous component of the human sensorimotor experience, or is does this propensity develop as we gain experience in the world; i.e., throughout childhood? Children with autism tend to express hyper-rigid behavioral patterns, including movement behaviors (rocking and hopping) and compulsive adherence to daily rituals. It might be interesting to explore if these children express similar flexibility of attention to chaotic motion.

The current work intends to provide an interesting observation for the benefit of the complexity theorist. We have identified not only that persons are sensitive to the dynamics of a chaotic oscillator, but in some ways have a particular preference to their dynamics. Further work will focus on how this approach may be useful in understanding behavioral coordination in a dynamic world rich with complex, and often chaotic, dynamics. This study provides solid ground from which to continue the investigation of sensorimotor coupling in response to a full spectrum of visual stimulus motion structure; from periodic, through chaos, to aperiodic.

## Data Availability Statement

The raw data supporting the conclusions of this manuscript will be made available by the authors, without undue reservation, to any qualified researcher.

## Ethics Statement

This study was approved and the protocol was carried out in accordance with the recommendations of the University of Nebraska Medical Center Institutional Review Board. All subjects gave written informed consent in accordance with the Declaration of Helsinki.

## Author Contributions

JH and NS contributed to the conception and design of the study. JH organized the database, performed the statistical analysis, and wrote the first draft of the manuscript. Both authors contributed to data interpretation, manuscript revision, and approval of the submitted version.

## Conflict of Interest Statement

The authors declare that the research was conducted in the absence of any commercial or financial relationships that could be construed as a potential conflict of interest.
